# Elite controllers: A heterogeneous group of HIV-infected patients

**DOI:** 10.1080/21505594.2020.1788887

**Published:** 2020-07-22

**Authors:** María A. Navarrete-Muñoz, Clara Restrepo, José M. Benito, Norma Rallón

**Affiliations:** a HIV and Viral Hepatitis Research Laboratory, Instituto De Investigación Sanitaria Fundación Jiménez Díaz, Universidad Autónoma De Madrid (IIS-FJD, UAM), Madrid, Spain; b Hospital Universitario Rey Juan Carlos, Móstoles, Spain; c Biotechvana, Scientific Park, Madrid, Spain

**Keywords:** Functional cure, elite controllers, heterogeneity, virological control, immunological control, clinical outcomes

## Abstract

The exceptional group of ECs has been of great help, and will continue to provide invaluable insight with regard to reach a potential functional cure of HIV. However, there is no consensus on the immune correlates associated to this EC phenotype which preclude reaching a potential functional cure of HIV. The existing literature studying this population of individuals has indeed revealed that they are a very heterogeneous group regarding virological, immunological, and even clinical characteristics, and that among ECs only a very small proportion are homogeneous in terms of maintaining virological and immunological control in the long term (the so-called long-term elite controllers, LTECs). Thus, it is of pivotal relevance to identify the LTECs subjects and use them as the right model to redefine immune correlates of a truly functional cure. This review summarizes the evidence of the heterogeneity of HIV elite controllers (ECs) subjects in terms of virological, immunological and clinical outcomes, and the implications of this phenomenon to adequately consider this EC phenotype as the right model of a functional cure.

## Introduction

HIV elite controllers (ECs) are exceptional individuals who can control the virus replication using their own immune system in the absence of combination antiretroviral therapy (cART). This characteristic has made them a focus of interest since they could represent a model for a potential functional therapeutic cure (long remission of HIV viral load without the need for continuous treatment in patients who do not control the virus spontaneously) [1,2].

**Figure 1.**
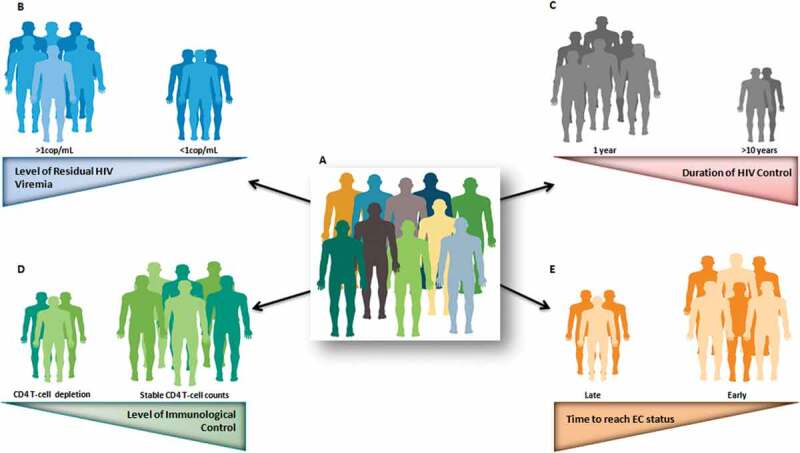
Heterogeneity observed in EC subjects (a), highlighting the main factors that can explained it: Level of residual HIV viremia (b); Duration of HIV control (c); Level of immunological control (d); Time to reach EC status (e). Colors in each individual represent the EC heterogeneity. Number of individuals represents the frequency of event in the EC population. Triangles indicate the magnitude of each factor with the narrow end representing the smaller amplitude and the wide end the larger.

However, beyond this exceptional control exhibited by this group of patients, there are some host and virus characteristics that are not shared by all ECs. Differences in genetic or immunological characteristics and clinical manifestations, as well as variation in virological characteristics, have been reported from different EC cohorts all around the world. This turns this unique population of patients into a heterogeneous group, making it difficult to justify placing them within a single category. All EC patients might be controlling viral load, but do they all have HIV viral load below the detection threshold of the most sensitive assays?; do they all have viral blips?; are they all able to maintain stable CD4 counts over time?; do they all experience progression events?; have they all experienced non-AIDS-related morbidities?; are they all long-term controllers? (Figure 1). In this review, we address the answers to these and other questions, together with the underlying factors related to that heterogeneity, which are of utmost relevance for the clinical management of these patients, as well as for considering these exceptional individuals as a model of a functional cure.

### Heterogeneity in EC definitions

The definition of EC, also known as “elite suppressors” or “HIV controllers”, which is based on undetectable viral load by standard assays, differs between studies since it depends on the plasma HIV-RNA threshold used [3–5] and the number of allowed blips over the follow-up period. For most studies, the duration of spontaneous control is long, but the range is wide, from few months to more than a decade [6], and the length of spontaneous control has been linked to the control of immune deficiency [1].

Perhaps the most commonly employed definition is the one proposed by the International HIV Controllers Consortium, which defines EC subjects as ART-naïve HIV-infected individuals with at least three plasma HIV-RNA determinations below 50–75 copies/mL for at least 1 year [3]. Other research groups have used adaptations of this definition with some variations in the plasma HIV-RNA threshold and length of follow-up, as described by Gurdasani *et al*. in a systematic review of definitions of EC and other extreme phenotypes [6]. All EC definitions included HIV-RNA thresholds ranging from 40 to 500 copies/mL, although the most frequent viral load threshold was 50 copies/mL, and some definitions allowed occasional viral load “blips” above the level of detectability. Regarding thresholds for the length of follow-up, this varied from 6 months to 16 years, and the most frequent follow-up threshold was 1 year [6]. Thus, the most agreed definition of an EC subject could be an HIV-infected patient followed-up for >1 year, naïve for cART, and with more than 90% of the plasma HIV-RNA measurements below 50 copies/mL.

However, none of these definitions include the ability to maintain stable CD4 + T-cell counts and, although most ECs show negligible rates of CD4 decrease over time, some of them experience progressive and significant CD4 T-cell depletion [7–11]. This point is important since studies considering ECs as a model for a functional cure must focus not only on the defining mechanisms of virological but also of immunological control, and for that, it is necessary to take into account those characteristics that make some ECs “*truly ECs”*. These characteristics could be those applied to the recently defined subgroup of EC termed LTECs (long-term elite controllers) [1], an extreme phenotype that might represent the best current model for a functional cure in whom both the virus and the immune deficiency are controlled. Thus, the definition of LTECs includes HIV-RNA viral load below 50 copies/mL for at least 10 years, with normal CD4 counts and a positive or null CD4 slope [12].

Heterogeneity in EC definitions between studies might hamper the search for correlates of viral control or immune protection in these individuals. However, these different definitions might be useful to better characterize the heterogeneity of EC phenotypes and, therefore, be used as a tool to carefully select the EC phenotype according to which aspect of disease control the research is focused.

### Heterogeneity in the level of residual HIV plasma viremia

The use of single-copy assays has revealed that ECs are heterogeneous with respect to residual plasma viremia. Multiple studies have found the existence of low-level plasma viremia in the majority of EC patients, with only a minor subset presenting persistent undetectable viremia using ultrasensitive assays [8,13–16]. Levels of plasma viremia using these assays were found to be higher and displayed a broader range in EC compared to patients on long-term cART [14,15]. Moreover, longitudinal measurements revealed a broad fluctuation of residual plasma viremia in the majority of EC patients [13].

Once it is established that ECs are heterogeneous in their ability to decrease plasma viremia to very low levels, the question that immediately arises is whether this heterogeneity is associated with the risk of HIV disease progression. Two previous studies addressing this issue have found that the level of residual plasma viremia was associated with both immunologic and virologic progression [8,13]. In one of these studies, analyzing a cohort of 77 EC with a median follow-up of 4 years, the CD4 counts declined was significantly higher in patients with levels of residual viremia above the limit of detection (1 copy/mL) compared to those with residual viremia <1 copy/mL [13]. In the other study, in a large cohort of 217 EC patients with a median follow-up of 5 years, virologic and/or immunologic progression was associated with higher levels of residual plasma viremia at the moment of inclusion in the cohort [8]. Implementation of single-copy assays in clinical practice is needed in order to validate these results in prospective studies with large cohorts of EC patients and could help to improve clinical management of this special population of patients.

Another important question arising from these observations is whether the range of residual plasma viremia observed in EC patients is the consequence of different levels of immune control exerted by different EC subjects. Answering this question is relevant for the search of immunological correlates of HIV control, and only those ECs achieving the strongest immunological control and the lowest level of residual plasma viremia should be considered as candidates for studies aimed at defining the best correlates of immune-mediated HIV control in the search for a functional cure.

### Heterogeneity in time to reach EC status

There is no conclusive evidence about how early HIV replication control is established after acute infection in EC patients and, more importantly, how broad is the time frame to reach viral replication control among different EC patients. This is largely due to the difficulty of identifying HIV-patients at the time of primary infection and/or seroconversion; conditions that are necessary to estimate the period between HIV infection and spontaneous control of viral replication. Two different approaches have been performed to address this issue, one based on large cohorts of seroconverters (patients for whom the date of seroconversion can be estimated), and another based on smaller cohorts of patients diagnosed at the time of primary/acute infection (before seroconversion). The controversial findings from multiple studies using these approaches suggest that the establishment of HIV replication control after acute infection is not homogeneous in all EC patients.

Multiple studies with seroconverter cohorts have reported different median delay to reach spontaneous viral control. Interestingly, the length of EC status was associated with the delay period to reach viral control [11,17,18], suggesting that shorter delays in reaching EC status increased the probability to maintain viral control for longer periods. On the other hand, studies performed in patients with primary/acute HIV infection have reported that most HIV controller patients reach control early after acute infection with a median delay of less than 1 year [19] or 6 months [20] (Table 1).

**Table 1.** Studies addressing the time-to-reach EC status.

Seroconverter Cohorts	Number of patients in the cohort	EC patients	% EC	Median delay to reach virological control (months)	Virological control period (years)	References
CASCADE	9896	140/9896	1.4	17	>5	[11]
	2176	145/2176	6.7	23	<1	[17]
US Department of Defense HIV Natural History Study	4621	17*/4621	0.4	12	2.3**	[18]
Primary/Acute HIV infection Cohorts	Number of patients in the cohort	EC patients	% EC	Median delay to reach virological control (months)	Virological control period (years)	References
ANRS CO06 PRIMO	211	8/211	3.8	6.2	>1	[20]
AIEDRP network	98	6/98	6.1	6.6	-	[19]

*25 EC patients were identified in this cohort, of which 17 had a known date of seroconversion ** Median of virological control duration (years), including all elite controllers, not only seroconverts (n = 25).

The factors influencing how early HIV replication control is established in ECs are heterogeneous and likely involve both viral and host factors: Miura *et al*. reported transmission of attenuated viruses in the majority of EC analyzed [19]; whereas Goujard *et al*. found that lower viral loads and higher CD4 counts during acute infection were associated with shorter time to reach complete virological control [20]. Moreover, it has been suggested that the viral control is caused by an active host response during the earliest stages of the infection [21]. Thus, it seems that the presence of effective host mechanisms from the beginning of infection is necessary to shorten the time needed to reach a complete suppression of viral replication.

### Heterogeneity in the duration of virological control

Another important question when studying the phenomenon of spontaneous control of HIV replication is how long the viral control is maintained once it is established. The question assumes that viral control is not indefinitely maintained and that a proportion of EC patients lose virological control at some point during follow-up.

Several studies have revealed that there is a large heterogeneity regarding this issue among different cohorts of EC patients, observing some differences between studies performed in seroconverters or primary/acute infection patients, and studies performed in patients with chronic infection [4,7,11,12,17,18,20,22]. Two studies with seroconverts EC patients found a period of virological control shorter than 3 years [17,18]. A similar result has been reported in a cohort of primary/acute-infected patients [20]. However, other studies performed in seroconverts patients have observed longer duration of virological control (>10 years) [4,11]. On the other hand, studies analyzing cohorts with chronic HIV-infected patients have also found longer periods of control. Two different studies reported a median period of control of 6 years [7,22], while another study observed a duration of virological control of 10 years [12] (Table 2). Some patients have shown an exceptional natural virological control for more than 25 years [23].

**Table 2.** Studies addressing the duration of virological control.

Seroconversion or Primary/acute HIV infection						
Cohort	HIV-patients included	EC patients	% EC	Virologic control period (years)	Median follow-up (years)	References
Centre Hospitalo- Universitaire de Bicetre and SEROCO-HEMOCO	2851	15/2851	0.6	10	10	[4]
CASCADE	9896	140/9896	1.4	85% EC >13	13.3	[11]
	2176	145/2176	6.7	0.9	6.6	[17]
US Department of Defense HIV Natural History Study	4621	17*/4621	0.4	2.3**	7.8	[18]
ANRS CO06 PRIMO	211	8/211	3.8	4.1	5.8	[20]
Chronic HIV infection						
Cohort	HIV-patients included	EC patients	% EC	Virologic control period (years)	Median follow-up (years)	References
Evelyn Jordan Center, Baltimore Veterans Administration (VA), and Maryland General Hospital	-	40	-	6.7	14	[21]
ECRIS	13,371	204/13,371	1.5	6.2	14.9	[7]
ANRS	46,880	69/46,880	0.15	>10	>10	[12]

*25 EC patients were identified in this cohort, of which 17 had a known date of seroconversion ** Median of virological control duration (years), including all elite controllers, not only seroconverts (n = 25).

Several conclusions can be drawn from these results. First, there is a large heterogeneity in the length of viral control reported by the different studies and, although part of this might be the consequence of methodological issues (including criteria for EC definition and length of follow up, among others), it is clear that there is also an intrinsic variability among EC subjects. Second, the CASCADE cohort demonstrates that control of HIV replication soon after seroconversion is not infrequent, but it is transitory in the majority of subjects [17]. Third, only a very small proportion of patients (around 0.15%) can maintain replication control for long periods (longer than 10 years) [12], the so-called long-term elite controllers (LTECs). Clearly, this small subset of LTEC is most valuable as a model to be used in studies aimed to find correlates of virological control in the search for an HIV vaccine and/or a functional cure.

**Figure 2.**
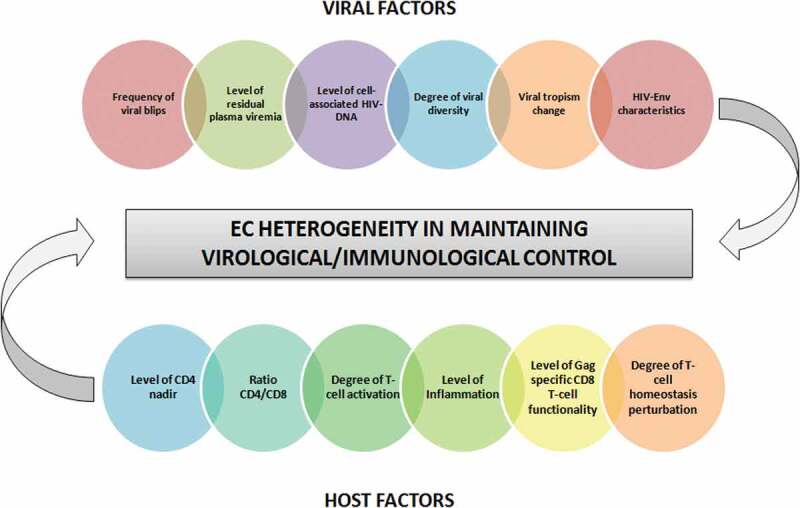
Factors determining the heterogeneity of EC patients in maintaining virological/immunological control.

The existence of variable periods of replication control in the majority of EC patients means that loss of control is reached at some time point during follow-up. Loss of virological control in EC has been documented by several authors [7,8,11,20,22]. Proportions of EC patients losing control have been as high as 50% [20] or as low as none of the patients losing control [4]. Differences in the criteria used to define EC status, in the length of follow-up, and in the criteria to define loss of virological control are in part responsible for this variation. However, there is an intrinsic variation among EC subjects in their ability to maintain suppression of HIV replication, and different risk factors of virological progression have been described, such as the frequency of viral blips during follow-up [8], levels of residual plasma viremia [8,11], levels of proviral HIV-DNA [8], HIV-Env characteristics [24], degree of viral diversity [25], and virus tropism change [26] (Figure 2). An episode of superinfection has also been linked to loss of control in some patients [20,22,26]. In contrast, as an example of the strong capacity of some individuals to suppress HIV, two different studies have reported on two EC patients maintaining long-term control of HIV and being able to control the second strain of HIV after an episode of superinfection [27,28]. A strong and sustained cellular and humoral immune response has been associated with this phenomenon [28].

An in-deep analysis of the immuno-virological and demographical factors associated with the risk of virological progression in EC patients has been recently performed, using a large cohort of EC patients from the Spanish AIDS Research Network elite controllers database (ECRIS): Among 204 patients meeting criteria of EC and with a median follow-up of 7.6 years, a rate of 23.5% of virological progression was observed. Risk factors for progression were sexual acquisition of HIV, low nadir of CD4 counts, high HIV viremia before inclusion as EC, and presence of hepatitis C co-infection [7]. A very original contribution of this study is the description of a clinical score based on these variables to estimate the individual risk of virological progression at 1, 5, and 10 years of follow-up [7].

### Heterogeneity in the loss of immunological control

Although the EC phenotype is not defined by CD4 counts, the majority of EC patients maintain relatively stable CD4 counts over time [17,18,20]. However, a variable proportion of ECs experience immunological progression (significant decline of CD4 T cells), with some of them reaching very low levels of CD4 counts and even AIDS-defining illnesses [26,29,30]. Moreover, the proportion of EC patients showing immunological progression is highly variable among studies, likely due to the design of the study (either cross-sectional or longitudinal), the length of follow-up, and the different criteria used to define immunological progression, among others (Table 3).

**Table 3.** Studies addressing the loss of immunological control.

Cohort	HIV-patients included	EC patients	% EC	Virological control period (years)	% EC with immune control loss	Cause	References
**CASCADE**	2176	145/2176	6.7	0.9	3.4	-	[17]
**ANRS CO06 PRIMO**	211	8/211	3.8	4.1	0	-	[20]
**US Department of Defense HIV Natural History Study**	4621	17/4621	0.4	2.3	4	-	[18]
**ANRS EP36**	34,317	81/34,317	0.3	10	62.3	Viral blips	[10]
**Boston hospitals**	-	90	-	3.6	8.9	High residual plasma viremia	[13]
**CO18/CO21 CODEX**	-	217	-	5	4.6	High residual plasma viremia, HIV-DNA, cell activation, viral blips and lower CD4 nadir	[8]
**ECRIS**	13,371	204/13,371	1.5	6.2	44.1	Sexual acquisition of HIV and lower CD4 nadir	[7]

Two different studies (using SCOPE and ANRS EP36 cohorts) have reported an important number of EC patients (as high as 10%) with very low levels of CD4 count at the moment of inclusion [10,29]. However, studies with a longitudinal design and with regular measurements of CD4 counts during the follow-up of EC patients have been more informative. Some studies have found that CD4 remains relatively stable as long as viral suppression is maintained [17,18,20], whereas others have found significant CD4 loss and progression to a certain threshold of immune deficiency, considering a negative CD4 counts slope as an indicator of immunological progression [10,13]. On the other hand, other studies have established a certain threshold of CD4 decrease as the criteria to define immunological progression in EC patients: 1) fall below 350 cells/µL or a decline of more than 200 cells/µL from the immediately preceding measurement [8]; and 2) decrease in CD4 counts during follow-up of at least 25% of the baseline CD4 count [7].

Regarding the mechanisms underlying loss of immunological control in EC patients, host and virus factors have been proposed (Table 3 and Figure 2). Among virus factors, the existence of persistent residual plasma viremia [8,13], the frequency of viral blips [8,10], the level of cell-associated HIV-DNA [8], a high viral diversity [25], and a change of viral tropism from CCR5 to CXCR4 [26] have all been associated with the probability of losing immunological control. Among host factors, a low nadir of CD4 [7,8,31], a decreased CD4/CD8 ratio [31], increased T-cell activation [8,26,29] and inflammation [8,25], and decreased Gag-specific CD8 + T-cell polyfunctionality [25,26] have been associated with the probability of immunological progression. Finally, our group has recently reported the existence of an important perturbation of T-cell homeostasis in EC patients experiencing immunological progression despite long-term undetectable viremia, suggesting that active pathogenic mechanisms are still present in some EC individuals [32].

### Heterogeneity in clinical outcomes

Although elite controllers can maintain control of viral replication and high CD4 counts in the absence of therapy, several studies have reported increased frequency of morbidity and mortality in this population of HIV-patients when compared to an HIV-uninfected population and HIV non-controller patients under cART (reviewed in ref. [33]). Loss of virological and/or immunological control in ECs might increase the risk of developing clinical events [10,17,29]. More strikingly, progression to AIDS and other clinical outcomes can also occur in the presence of undetectable HIV replication and high CD4 T-cell counts [17,20,29,34].

Among the different clinical events reported in EC, AIDS-defining pathologies are rare, as expected in a population of patients with good virologic and immunologic status [7,18,35]. Although these pathologies have been mainly reported in EC patients losing virologic and/or immunologic control [17,35], some authors have reported AIDS-defining conditions in ECs maintaining virologic suppression [7,17,29]. Much more frequent in EC patients are the clinical conditions that do not define AIDS, the so-called non-AIDS defining events (nADEs) [34–37]. Cardiovascular diseases [34] atherosclerosis [36,37], and cancers [35] are among the most common nADEs reported in ECs. Interestingly, high levels of persistent inflammation markers found in ECs have been associated with an increased risk of developing nADEs [37].

Multiple studies have longitudinally analyzed the incidence rate of clinical outcomes in EC patients compared to either untreated or treated non-controller patients [7,18,34,38,39]. Overall, there is great heterogeneity in the incidence rates reported in these studies, most likely due to differences in clinical outcome analyzed (AIDS-defining events versus nADES), and comparison groups included (viremic controllers, untreated non-controllers, treated controllers, healthy subjects). Two previous studies have found low incidence rates of AIDS or death in ECs [7,18]. One of these studies analyzed a small cohort of ECs and found a lower incidence of AIDS and death compared to a group of non-controller-untreated patients [18]. The other study analyzed a large cohort of ECs with a median follow-up of 7 years and found very low incidence rates of AIDS or death [7].

Incidence rates of nADEs have been analyzed in two different studies [38,39]. Both studies have reported a similar incidence of nADEs in EC, although in one of them the incidence in EC was similar to that in non-controller patients [38], whereas in the other study EC patients showed a lower incidence compared to the non-controller patients [39]. This difference is likely due to the criteria used to select the comparison group of non-controller patients. In the study by Lucero *et al*., a CD4 count higher than 500 cells/µL was an inclusion criterion, and the majority of non-controller patients were receiving cART [38]. In contrast, the study by Dominguez-Molina *et al*. included non-controller patients irrespective of CD4 count and treatment status [39]. Interestingly, the study by Dominguez-Molina *et al*. showed that the incidence of nADEs increased in EC patients that lose virological control, mainly due to an increase in cardiovascular events. Lastly, Crowell *et al*. have reported higher rates of hospitalization among EC patients compared to non-controller patients on cART, with cardiovascular events being the most frequent nADEs [34]. The conclusion from these studies is that the incidence of nADEs in ECs is as high as that observed in non-controller patients with cART-mediated control of HIV replication, but lower than in patients with uncontrolled viral replication. However, some clinical conditions, such as cardiovascular events, seem to be more prevalent in ECs than in treated non-controller patients.

## Concluding remarks

Among the total population of HIV-infected patients, ECs seem to be the closest model to a functional cure. For this reason, they have been considered as a population of reference to study factors involved in the ability to control both HIV replication and HIV-induced immunodeficiency. There is consensus that ECs can give us clues in the search for therapeutic strategies aimed to achieve this state in the general population of patients and more importantly in the search for surrogates of protection in vaccine trials. However, it has become clear that “not all HIV-elite controllers are created equal” and, except for the basic operational definition of elite controller status, there is a large heterogeneity in terms of virological, immunological, and clinical outcomes in these patients. The acknowledgment of this fact is of paramount importance since that will lead us to pin down the best model of functional cure in which to concentrate our efforts in the search for therapeutic strategies aimed at a cure for HIV.

## Figures and Tables

**Table:** 

cART	combination antiretroviral therapy
AIDS	acquired immune deficiency syndrome
HIV	human immunodeficiency virus
DNA	deoxyribonucleic acid
RNA	ribonucleic acid
EC	elite controller
LTEC	long-term elite controller
CD4	cluster of differentiation 4
CD8	cluster of differentiation 8
nADEs	non-AIDS defining events

## Search strategy and selection criteria

Relevant scientific literature was surveyed to review evidence and prepare the manuscript. We searched PubMed for English language papers published between October 2005 and May 2020. Search terms included “Elite controller”, “HIV controller”, “Extreme phenotype HIV”, “LTNP-EC”, “Morbidities and Elite controller”, “EC and loss of HIV control”, “EC and loss of immunological control”, and “HIV functional cure”. Two authors (MANM and CR) screened abstracts for relevance and reviewed full-text articles deemed relevant to the topics address in the manuscript.
